# Structurally distinct mitoviruses: are they an ancestral lineage of the *Mitoviridae* exclusive to arbuscular mycorrhizal fungi (Glomeromycotina)?

**DOI:** 10.1128/mbio.00240-23

**Published:** 2023-05-10

**Authors:** Tatsuhiro Ezawa, Alessandro Silvestri, Hayato Maruyama, Keitaro Tawaraya, Mei Suzuki, Yu Duan, Massimo Turina, Luisa Lanfranco

**Affiliations:** 1 Graduate School of Agriculture, Hokkaido University, Sapporo, Japan; 2 Department of Life Sciences and Systems Biology, University of Torino, Torino, Italy; 3 Faculty of Agriculture, Yamagata University, Tsuruoka, Japan; 4 Institute for Sustainable Plant Protection–CNR Torino, Torino, Italy; Friedrich-Schiller-Universitat, Jena, Germany

**Keywords:** mycoviruses, mycorrhizae, *Mitoviridae*, Glomeromycotina, evolution, plus-strand RNA virus, soil microbiology

## Abstract

Mitoviruses in the family *Mitoviridae* are the mitochondria-replicating “naked RNA viruses” with genomes encoding only the replicase RNA-dependent RNA polymerase (RdRp) and prevalent across fungi, plants, and invertebrates. Arbuscular mycorrhizal fungi in the subphylum Glomeromycotina are obligate plant symbionts that deliver water and nutrients to the host. We discovered distinct mitoviruses in glomeromycotinian fungi, namely “large duamitovirus,” encoding unusually large RdRp with a unique N-terminal motif that is endogenized in some host genomes. More than 400 viral sequences similar to the large duamitoviruses are present in metatranscriptome databases. They are globally distributed in soil ecosystems, consistent with the cosmopolitan distribution of glomeromycotinian fungi, and formed the most basal clade of the *Mitoviridae* in phylogenetic analysis. Given that glomeromycotinian fungi are the only confirmed hosts of these viruses, we propose the hypothesis that large duamitoviruses are the most ancestral lineage of the *Mitoviridae* that have been maintained exclusively in glomeromycotinian fungi.

## OPINION/HYPOTHESIS

Mitochondria, beyond their role in energy generation, are central to several fundamental cellular processes, e.g., calcium homeostasis and redox balancing, and also harbor unique viruses, namely mitoviruses in the family *Mitoviridae*. Mitoviruses were likely to evolve from leviviruses in the family *Leviviridae*, RNA bacteriophages, by losing the capsid protein, which yielded mitochondria-replicating “naked RNA replicons” that encode only the replicase RNA-dependent RNA polymerase (RdRp) (1). They were first discovered in a hypovirulent strain of the plant pathogenic fungus *Cryphonectria parasitica* (2). Decades of extensive surveys have revealed that mitoviruses are prevalent not only in fungi but also across plants (3) and invertebrates (4). Although knowledge of their impact on the host is limited, the involvement in the hypovirulence of plant pathogens (2) and plant resilience to abiotic stress via activating metabolic processes (5) has been proposed. Mitoviruses are transmitted vertically to progeny through cell division and sporulation, and horizontally to different individuals via hyphal fusion (6). However, the fact that closely related mitoviruses have been identified across distant fungal phyla (7) and even across the kingdoms Fungi and Plants (3) suggests that cross-phylum/kingdom horizontal transfer also occurs. Arbuscular mycorrhizal fungi in the subphylum Glomeromycotina are an early-diverging lineage that drove the terrestrialization of early plants 400 million years ago (8) by delivering water and mineral nutrients to the host (9). These fungi harbor a range of viruses and, so far, 11 mitoviruses have been described in four host species (10 - 12), in addition to two mitoviruses deposited in the database but not yet published in a peer-reviewed paper. Here, we report a structurally distinct group of mitoviruses that forms the most basal clade in the *Mitoviridae*. So far, they have been found only in glomeromycotinian fungi, which raises the question of whether they are an ancient lineage that has been maintained exclusively in glomeromycotinian fungi.

## STRUCTURAL UNIQUENESS

**Fig 1:**
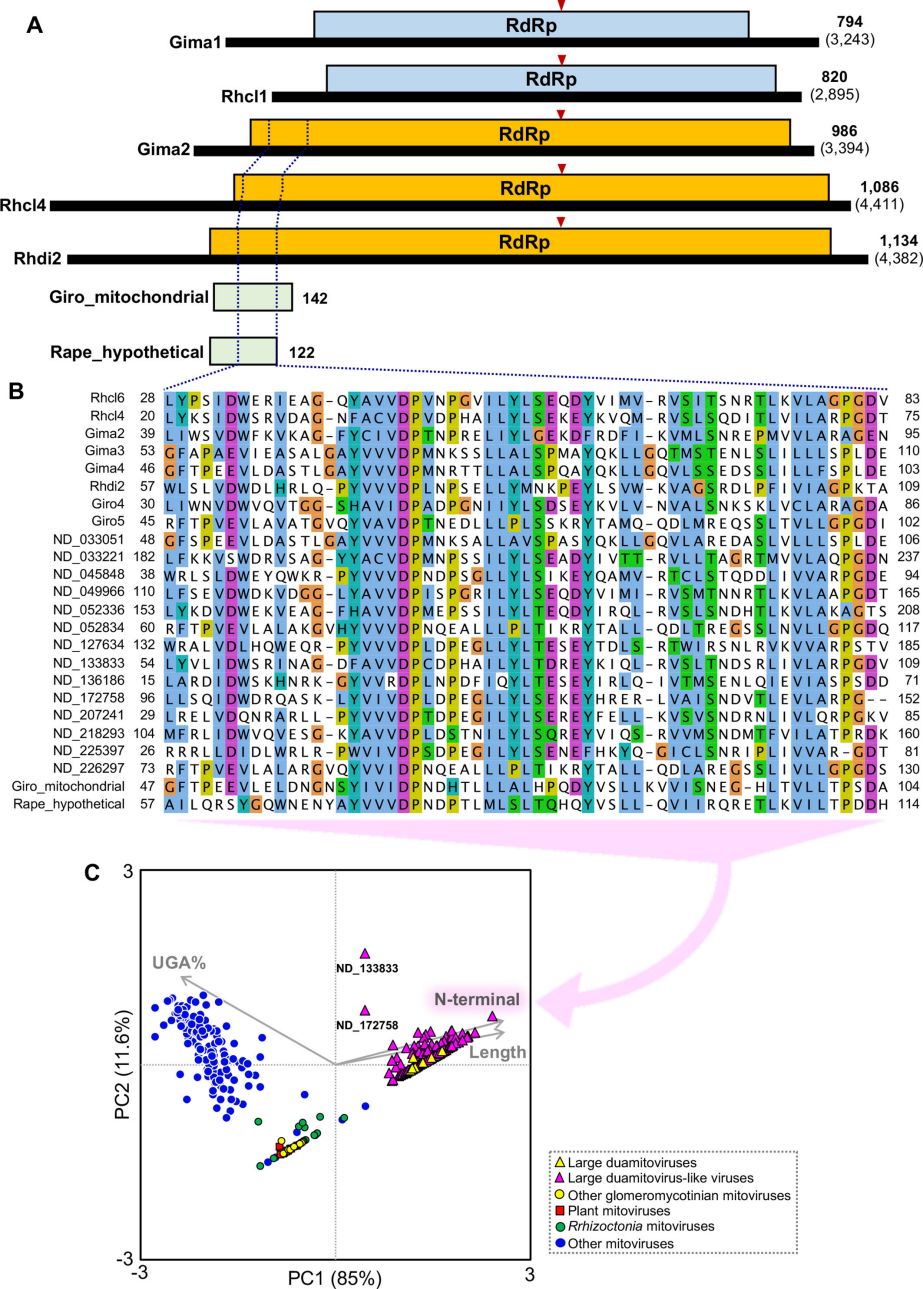
Structural characterization of glomeromycotinian mitovirus genomes that encode large RNA-dependent RNA polymerase (RdRp). (**A**) Genome structure of glomeromycotinian duamitoviruses that encode normal (light blue boxes) and large (orange boxes) RdRp and hypothetical proteins (light green boxes) encoded by *Gigaspora rosea* mitochondrial genome (Giro_mitochodrial) and *Racocetra persica* nuclear genome (Rape_hypothetical). Positions of N-terminal motif is indicated with dotted lines. The RdRp sequences are aligned at the GDD motif (red arrow heads, Fig. S1). The numbers of amino acid (bold letters) and nucleotide (in parentheses) residues are indicated on the right. (**B**) Multiple alignment of N-terminal motif of glomeromycotinian large duamitoviruses, large duamitoviruses-like viruses found in the RNA Viruses in Metatranscriptomes database (ND series), and hypothetical proteins encoded by the fungal genomes. The amino acid sequences were aligned with Clustal W implemented in MEGA X, and shared residues are highlighted with colors in Jalview. (**C**) Structural characterization of the large duamitovirus-like viruses found in the public databases (Dataset S2) by principal component analysis in reference to the glomeromycotinian large duamitoviruses and the 105+94 reference species collection listed in the International Committee on Taxonomy of Viruses (ICTV) report (Dataset S1). The presence/absence of the N-terminal motif (N-terminal), aa length of RdRp (length), and percentage of UGA codon (UGA%) are employed as factors. Abbreviations of the viral names and their accession numbers are listed in Datasets S1 (duamitoviruses) and S2 (ND series).

We here identified 10 new glomeromycotinian mitoviruses; five, two, and three from *Gigaspora rosea* DAOM194757, *Rhizophagus clarus* CK001, and *R. clarus* OL1, respectively (Text S1 and Dataset S1), showing the conserved mitovirus RdRp motif (pfam05919) (Fig. S1). A preliminary Blastp search against the 105 reference species proposed for mitovirus taxonomy in the International Committee on Taxonomy of Viruses (ICTV) report (13) indicated that the 10 new mitoviruses, in addition to the previously reported 13 glomeromycotinian mitoviruses (10 - 12), were distributed across the three genera: 2, 14, and 7 viruses in the *Unuamitovirus*, *Duamitovirus*, and *Triamitovirus*, respectively (Dataset S1). One general feature of these glomeromycotinian mitoviruses is the predominant use of UGG codons for Trp, while UGA codons, which are prevalent in most mitoviruses (except for plant and *Rhizoctonia* mitoviruses), are rare (10 - 12, 14). Detailed structural analysis of these mitoviruses revealed that 8 of the 14 duamitoviruses encode unusually large RdRps of 986–1,134 aa (1,036 aa on average), compared with those of the 105+94 reference species (i.e., the sum of the 105 and 94 additional reference species in the ICTV report) of which RdRps range from 556 to 1,136 aa (761 aa on average). Furthermore, these large RdRps share a unique N-terminal motif of 53–58 aa, which was also found in hypothetical proteins encoded by the genomes of the glomeromycotinian fungi *G. rosea* and *Racocetra persica* (Fig. 1A and B). Based on these results, we defined the viruses that encode RdRp of 900 aa or longer with the N-terminal motif as “large duamitoviruses.” To explore their distribution, we searched viral sequences that encode large RdRps with the N-terminal motif both in the GenBank database and in the RNA Viruses in Metatranscriptomes (RVMT) (Text S1), from which hundreds of new mitoviruses have recently been discovered (15, 16). In total, 37 (GenBank) and 415 (RVMT) large duamitovirus-like sequences (Dataset S2) were identified and, notably, all were found in the metatranscriptome samples from soil and roots that are the only ecological niches of glomeromycotinian fungi. In these processes, potential coding regions of the N-terminal motif, including different reading frames from RdRp, were also searched, but no potential reading frames, except for the RdRps of large duamitoviruses, were found in known viral genomes in the GenBank database. These 37+415 sequences encode RdRps of 902–1,445 aa (1,069 aa on average) in which UGA codons are rare (1.4% on average), with two exceptions, ND_133833 (65%) and ND_172758 (39%). Principal component analysis on these viral sequences was conducted with the three factors, presence/absence of the N-terminal motif, RdRp aa length, and percentage UGA codon, with respect to the 105+94 reference species. All the large duamitovirus-like sequences, except for ND_133833 and ND_172758, co-localized with the glomeromycotinian large duamitoviruses and clearly separated from the others (Fig. 1C), mirroring their structural uniqueness.

## ANCESTRAL LINEAGE

**Fig 2:**
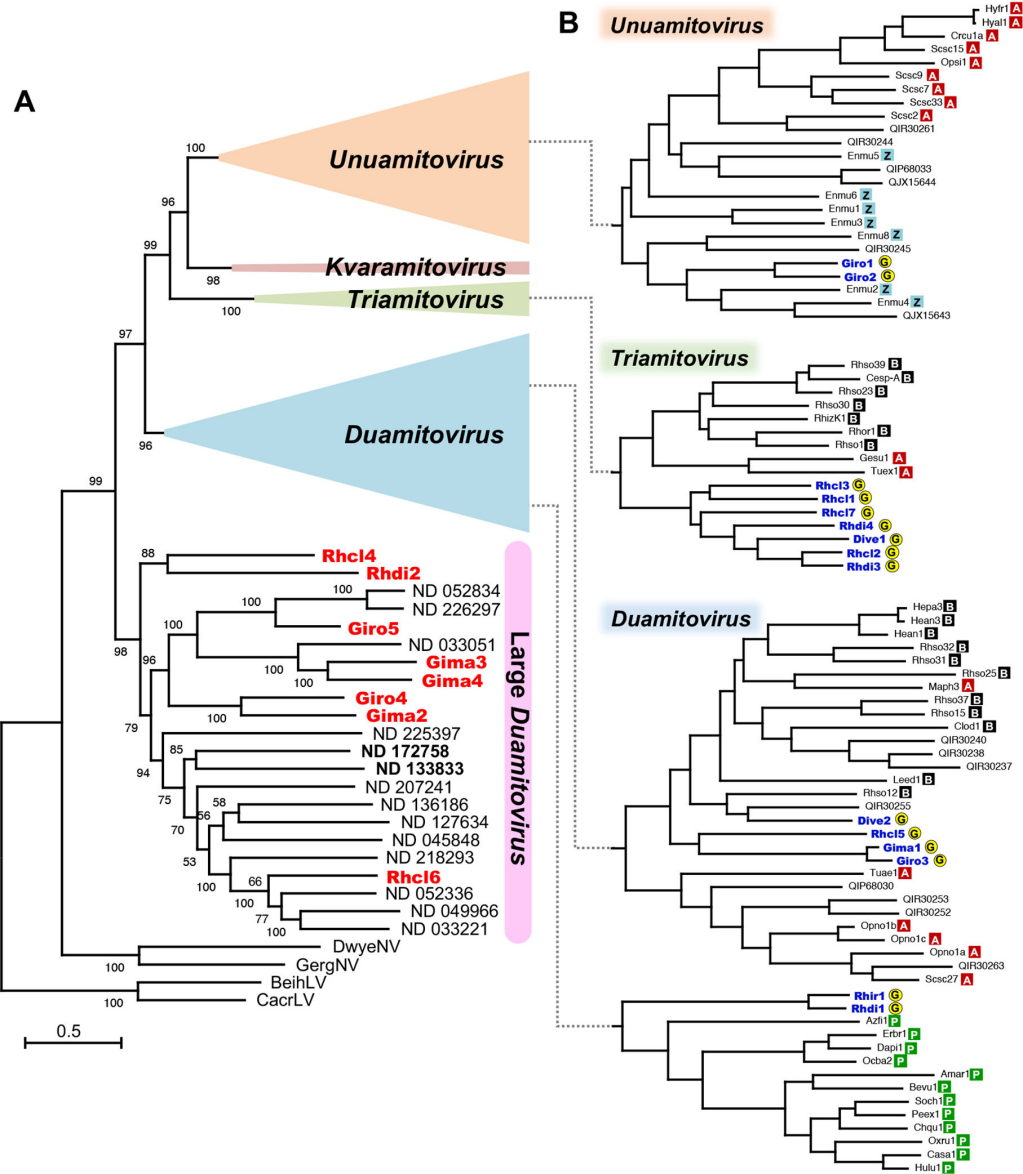
(**A**) Phylogenetic analysis of glomeromycotinian large duamitoviruses (red letters) and large duamitovirus-like viruses (black letters) with the 105+94 reference species (Dataset S1). The full-length amino acid sequences of RNA-dependent RNA polymerase were aligned with MAFFT, and the maximum-likelihood tree was inferred by IQ-TREE equipped with the ModelFinder (VT+F+I+G4 mixed model), rooting on the *Narnaviridae* (DwyeNV and GergNV) and *Leviviridae* (BhLV and CcLV). Branch support was tested by the UFBoot2 (1,000 replicates), and the values are shown in percentage. The genera *Unuamitovirus*, *Triamitovirus*, *Kvaramitovirus*, and the other clusters of *Duamitovirus* were compressed. Abbreviations of the viral names and their accession numbers are listed in Dataset S1. ND 133833 and ND 172758 indicated with bold letters are those that employed UGA codons for Trp at high frequencies (Fig. 1 and Fig. S2). (**B**) Branches of the maximum-likelihood tree to which other (non-large) glomeromycotinian mitoviruses (blue letters) belong. The phyla/kingdom of host species are indicated on the right of virus names: A, Ascomycota; B, Basidiomycota; G, Glomeromycotina; P, plant; Z, Zoopagomycota.

To investigate their phylogenetic position, the eight glomeromycotinian large duamitoviruses and 14 large duamitovirus-like sequences that vary in UGA-codon frequency (Dataset S2 and Fig. S2) were aligned with the 105+94 reference species using the MAFFT alignment program, and a maximum likelihood tree was inferred, following the method in the ICVT report (Text S1). All the glomeromycotinian large duamitoviruses formed the most basal clade of mitoviruses together with the 14 large duamitovirus-like viruses, including those that use UGA codons at high frequencies (Fig. 2A). This clade was robust, using up to 40 large duamitovirus-like sequences (Fig. S3), using Clustal Omega instead of MAFFT (Fig. S4), and even using only the evolutionarily conserved RdRp domain sequences (Fig. S5), suggesting that they represent an ancestral lineage in the *Mitoviridae*. In this clade, glomeromycotinian fungi are the only confirmed hosts, and the hosts of large duamitovirus-like viruses are also likely glomeromycotinian fungi, given their structural similarity, ecological niches, and phylogenetic positions. This is unusual for the other glomeromycotinian mitoviruses that have many close relatives harbored by distantly related fungi and plants (Fig. 2B). These results suggest that horizontal transfer of large duamitoviruses to distant eukaryotes did not occur in the past, which led us to examine the possibility of niche/habitat differentiation between the hosts of large duamitoviruses and other mitovirus hosts.

**Fig 3:**
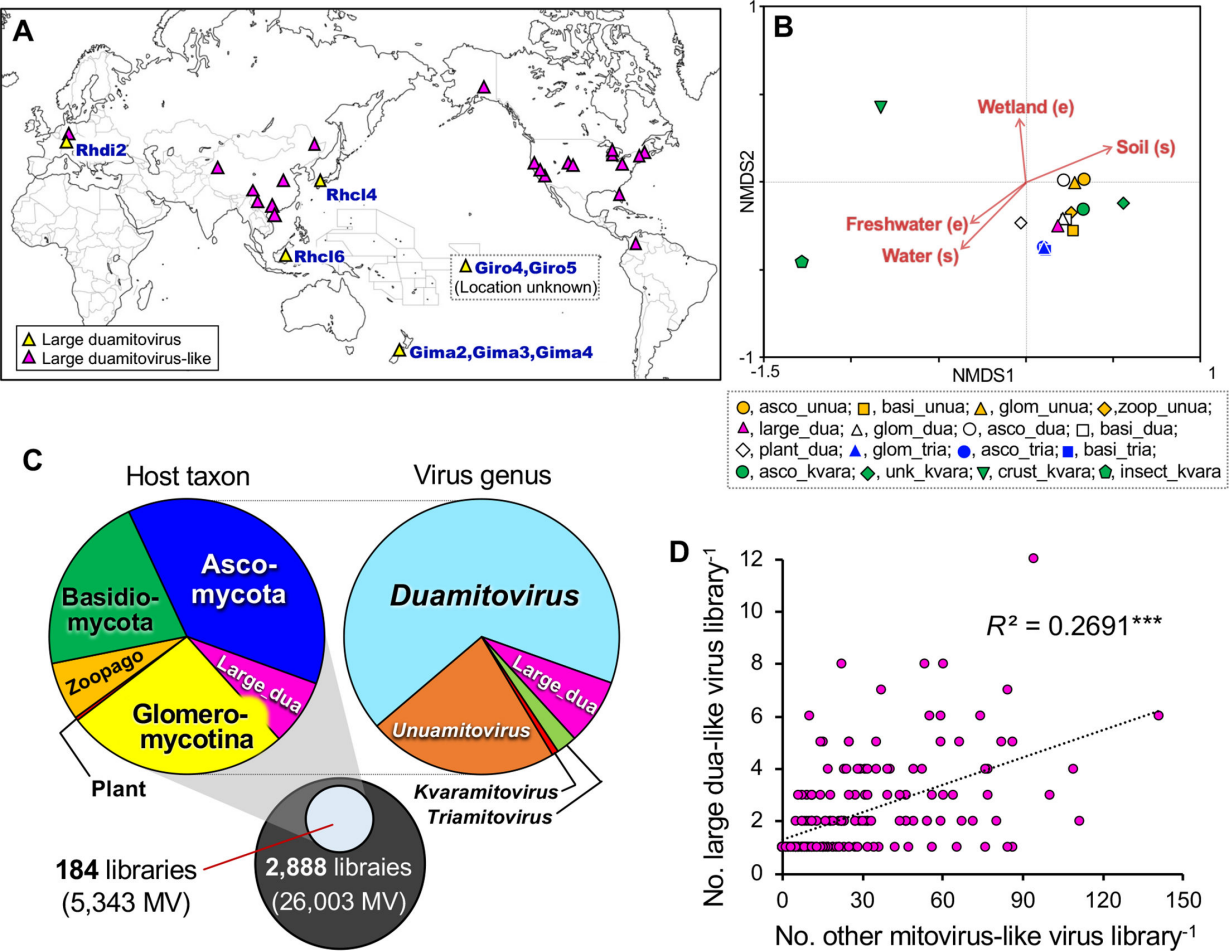
(**A**) Geographic distribution of the habitats of glomeromycotinian large duamitoviruses (yellow triangles) and large duamitovirus-like viruses (magenta triangles). The locations from which the host fungi (large duamitoviruses) and soil/root samples (large duamitovirus-like viruses) were collected are mapped (Datasets S1 and S2). The white map was obtained at www.freemap.jp. (**B**) A genus-host-taxon-environment biplot of non-metric multidimensional scaling (NMDS) of the mitovirus communities analyzed with the metadata in the RVMT database (2 dimensions, stress = 0.1295). Top 20 hits of the 105 reference species were searched, clustered at 100% identity (yielded 1,052 non-redundant sequences), grouped by putative genera, host phyla/domains, sampling location, ecosystem, and sample type (Dataset S3), and combined with the large duamitovirus-like sequences to construct a mitovirus-community dataset. Correlations of the NMDS1 and 2 scores with the frequencies of the ecosystem (**E**) and sample type (**S**) in the individual study sites were assessed in the vegan package in R, and those that showed significant correlations at *P* < 0.05 were indicated as red arrows. Abbreviations for putative host taxa are asco, Ascomycota; basi, Basidiomycota; glom, Glomeromycotina; zoop, Zoopagomycota; unk, unknown host; crust, Crustacea; insect, Insecta, and those for viral genera are unua, *Unuamitovirus*; dua, *Duamitovirus*; tria, *Triamitovirus*; kvara, *Kvaramitovirus*; large_dua-like, large duamitovirus-like viruses. (**C**) Putative host phyla/kingdom and virus genera of the mitovirus-like viruses co-existing with the large duamitovirus-like viruses (Large_dua) in the sequencing libraries. Among the 26,003 viral sequences in 2,888 libraries in the RVMT database, 5,343 sequences were extracted based on the similarity to the 105 reference species from the 184 libraries from which the 415 large duamitovirus-like viruses were identified. Host taxa and virus genera were assigned in reference to the top hit reference species. (**D**) Correlations of the numbers of large duamitovirus-like viruses and those of the other mitovirus-like viruses in the 184 sequencing libraries. ****, *P* < 0.001 (Student’s *t*-test).

## GLOBAL DISTRIBUTION AND NICHE OVERLAP

To explore the preferential niches/habitats of the hosts of large duamitoviruses, we first mapped the geographic locations from which the glomeromycotinian hosts of eight large duamitoviruses and the soil/root samples of the 37 (GenBank) + 415 (RVMT) large duamitovirus-like viruses were isolated. The large duamitoviruses and their related sequences were globally distributed, except for African and Australian continents of which virus-targeted metatranscriptome data are currently absent (Fig. 3A), and this pattern is consistent with the cosmopolitan distribution of glomeromycotinian fungi (17). From the *“Mitoviridae*” and “Unclassified” datasets in the RVMT, top 20 sequences that showed similarity to each of the 105 reference species and (non-large) glomeromycotinian mitoviruses were retrieved, and those that encode complete protein (putative RdRp) of 500 aa or longer were extracted (1,052 non-redundant sequences, Dataset S3). Then, these sequences were grouped by putative virus genera and host taxa in reference to the top-hit reference species and also by sampling location, ecosystem, and sample type and subjected to non-metric multidimensional scaling to explore environmental factors that determine their distribution (Text S1). The genus-host-taxon-environment biplot indicated that except for those similar to crustacean and insect kvaramitoviruses, the large duamitovirus-like viruses co-localized with a majority of other mitoviruses (Fig. 3B). Notably, most mitovirus-like viruses, including large duamitovirus-like viruses, were predominantly detected in soil samples, irrespective of their genera and host taxa, suggesting niche overlap among them. To confirm whether the hosts of large duamitoviruses-like viruses co-exist with other mitovirus hosts at the habitat scale, we inferred host taxa/virus genera detected in the same metatranscriptome sequencing libraries from which the large duamitovirus-like viruses were detected (Text S1). The *“Mitoviridae*” and “Unclassified” datasets in the RVMT contain 26,003 mitovirus-like (non-redundant) sequences that encode complete protein of 500 aa or longer in 2,888 sequencing libraries, including 8,361 sequences in the 184 libraries from which the 415 large duamitovirus-like sequences were identified. From the 8,361 sequences, those that showed significant similarity to the 105 reference species and (non-large) glomeromycotinian mitoviruses were extracted (5,343 sequences), and host taxa (phyla/kingdom) and virus genera were assigned to them in reference to the top hit reference species (Dataset S4). Although about one-third of all mitovirus-like viruses in the 184 libraries were putatively harbored by glomeromycotinian fungi (1,403 non-large mitoviruses and 415 large duamitoviruses), the others were likely harbored by a variety of eukaryotes: 2,003 by Ascomycota, 1,136 by Basidiomycota, 366 by Zoopagomycota fungi, and 21 by plants (Fig. 3C). Not only the hosts but also the viruses were also diverse; they were assigned across all the four genera: 1,196, 3,562, 130, and 40 viruses in the *Unuamitovirus*, *Duamitovirus*, *Triamitovirus*, and *Kvaramitovirus*, respectively. In addition, the numbers of large duamitovirus-like viruses and the other mitovirus-like viruses in each library were positively correlated (*P* < 0.001) (Fig. 3D). These results confirm that the hosts of large duamitovirus-like viruses, probably glomeromycotinian fungi, share habitats with diverse eukaryotes that harbor diverse mitoviruses and, further, that the abundance of large duamitovirus is higher where the overall population of mitoviruses is higher, implying that the niches/habitats of the hosts of large duamitoviruses are not differentiated between other mitovirus hosts, that is, there is no ecological barrier to constrain the horizontal transfer of large duamitoviruses to distant eukaryotes.

## ARE GLOMEROMYCOTINIAN FUNGI AN EXCLUSIVE HOST?

Viruses in plant symbiotic fungi have so far been largely unexplored. In ectomycorrhizal fungi that associate with trees, only three mitoviruses have been described in *Tuber excavatum* (Ascomycota) (18), *Geopora sumneriana* (Ascomycota) (19), and *Albatrellopsis flettii* (Basidiomycota) (20). One mitovirus was found in one of the two isolates of orchid mycorrhizal fungus *Ceratobasidium* sp. (Basidiomycota) (21). Recently, 37 ericoid mycorrhizal fungi (Ascomycota) that associate with ericaceous shrubs and 12 orchid mycorrhizal fungi (Basidiomycota) were examined, but only one mitovirus was identified in the orchid symbiont *Ceratobasidium* sp (22). Given the ubiquitous distribution of arbuscular mycorrhiza in terrestrial ecosystems, including forest, shrubland, and grassland, glomeromycotinian fungi share niches/habitats with all types of mycorrhizal fungi. Large duamitoviruses, however, have been identified in none of these mycorrhizal fungi, except for arbuscular mycorrhizal fungi in the Glomeromycotina. One might expect that more basal fungi would harbor large duamitoviruses, given that large duamitoviruses form the most basal clade of the *Mitoviridae*. In the *in silico* surveys on the public transcriptome data of the entomopathogenic fungus *Entomophthora muscae* that belong to the Zoopagomycota, a more basal phylum than the Glomeromycotina, eight mitoviruses were identified (23), but they all were assigned to the genus *Unuamitovirus* (Fig. 2B and Dataset S1). All these studies indirectly support the hypothesis that glomeromycotinian fungi are an exclusive host of large duamitoviruses.

Given the apparent niche/habitat overlap between the hosts of large duamitoviruses and the variety of mitovirus-harboring host eukaryotes, a more likely explanation for the absence of large duamitoviruses in other eukaryotes is that there is a transmission barrier between distant phyla/kingdoms. The structural uniqueness of large duamitoviruses, e.g., the N-terminal motif, could play a role in the restriction of their horizontal movement. Although translation of the motif sequence has not been confirmed, there is a general rule that translation starts at the first AUG codon, and if it is not translated or has no function, the sequence would not be conserved among the geographically isolated viruses, suggesting that the motif is functionally active. We can also speculate that large duamitoviruses are less competitive than “small mitoviruses” that require less resource for replication. Glomeromycotinian fungi have been cultured *in vitro* conventionally in association with root-organ cultures (24), but are recently found to be culturable without plant roots in the presence of fatty acid, at least for *R. clarus* and *R. irregularis* (25). The growth rates of these fungi, however, are quite slow, irrespective of the presence (24) or absence (25) of roots; it took 2 to 3 mo for sporulation, probably due to the nature of plant symbionts that acquire carbon rather modestly from the host to sustain host growth for securing their own survival. In such slow-growing organisms, viruses may replicate at a low rate due to resource limitations. Under these conditions, the replication efficiency is unlikely to critically affect competition among co-existing mitoviruses, enabling the survival of large duamitoviruses. In this context, large duamitoviruses could be distributed in other slow-growing eukaryotes. The differences in the codon frequency for Trp may not be a serious barrier for horizontal movement, at least among fungi, given that both UGA and UGG codons encode Trp in fungal mitochondrial genomes. The plant duamitoviruses and *Rhizoctonia* triamitoviruses that are closely related to some (non-large) glomeromycotinian mitoviruses employ no or few UGA codons for Trp (Fig. 2B and Dataset S1), as expected because these viruses were likely to extend their host ranges *via* horizontal transfer to or from the glomeromycotinian fungi. There are, however, exceptions; e.g., *E. muscae* mitoviruses 2, 4, and 8 in the *Unuamitovirus* use UGA codons at high frequencies (38–75% of Trp residues, Dataset S1), but are closely related to *G. rosea* mitoviruses 1 and 2 that employ UGA codons at 0 and 6%, respectively, suggesting that mitoviruses adapt their codon frequency flexibly and rapidly to that of the host genome.

## CONCLUSION

We demonstrated that glomeromycotinian fungi harbor structurally distinct mitoviruses, namely large duamitoviruses, that form the most basal clade in the *Mitoviridae*. They are globally distributed and abundant in soil ecosystems, but intriguingly absent in other eukaryotes that share niches/habitats with glomeromycotinian fungi, suggesting an inhibitory mechanism for horizontal transfer to other eukaryotes. This atypical host range of large duamitoviruses and their phylogenetic position led us to hypothesize that large duamitoviruses are the most ancestral lineage maintained exclusively in glomeromycotinian fungi. To test this hypothesis, several issues should be addressed. It is difficult to provide direct evidence of the “absence” of the viruses in other eukaryotes; instead, the characterization of the viromes of new isolates/species of glomeromycotinian fungi would represent a solid, although laborious, approach to unveil the occurrence and distribution of large duamitoviruses. Exploring public transcriptomic data not only from fungal hyphae/spore but also from roots grown in the presence and absence of glomeromycotinian fungi may be a complementary and efficient tool for this purpose. In our survey, the N-terminal motif that illustrates the structural uniqueness of large duamitoviruses could not be found in the members of the closely related families *Leviviridae* (RNA bacteriophages) and *Narnaviridae* (“naked RNA viruses” replicate in the eukaryotic cytosol). Although the origin of this motif, as well as its biological function, deserves investigation, the two events of endogenization of the motif sequence suggest a long history of coevolution between glomeromycotinian fungi and large duamitoviruses. The possibility that a prototype of large duamitoviruses obtained the sequence from the fungal host can also be envisaged because glomeromycotinian fungi are, so far, the only organisms that possess the motif sequence in their genomes. Further surveys on fungal genomes/transcriptomes are necessary. Taken together, we provide new insights into the evolution and ecology of mitoviruses, showing that they are a highly diverse group of mycoviruses and shedding light on their unique evolution across the prokaryote-eukaryote domain boundary.

## SUPPLEMENTAL MATERIAL

Text S1 mbio.00240-23-s0001.pdf Detailed materials and methods for the identification of new mitoviruses, database surveys, and phylogenetic/ecological analyses with references.

Fig. S1 mbio.00240-23-s0002.pdf Multiple alignment of the conserved amino acid motifs in mitovirus RNA-dependent RNA polymerase in the newly identified glomeromycotinian mitoviruses. The RdRp sequences from the 10 newly identified viruses (indicated with asterisks and bold letters), previously described 5 glomeromycotinian viruses (bold letters), Duamitovirus crpa1, and Duamitovirus soch1 (Dataset S1) were aligned by Clustal W implemented in MEGA X. Aligned amino acid residues are highlighted with colors in Jalview, and the positions of motifs are indicated by black lines.

Fig. S2 mbio.00240-23-s0003.pdf Distribution of UGA codon for Trp (red arrow heads) in the RdRps of glomeromycotinian large duamitovirus (*G. rosea* MV5) and six large duamitovirus-like viruses employed for the phylogenetic analyses (Fig. S3 to S7). The RdRps are aligned at the GDD motif (purple boxes), and the N-terminal motif is indicated by blue boxes.

Fig. S3 mbio.00240-23-s0004.pdf Phylogenetic positions of glomeromycotinian large duamitoviruses (red letters) and 40 large duamitovirus-like viruses (ND series) with reference to the 105+94 species (B). The full-length amino acid sequences of RdRp were aligned with MAFFT (L-INS-i strategy), and the maximum-likelihood tree was inferred by IQ-Tree (VT+F+I+G4 mixed model) as described in Text S1. Kva, Kvaramitovirus.

Fig. S4 mbio.00240-23-s0005.pdf Phylogenetic positions of glomeromycotinian large duamitoviruses (red letters) and 14 large duamitovirus-like viruses (ND series) with reference to the 105+94 species. The full-length amino acid sequences of RdRp were aligned with Clustal Omega, and the maximum-likelihood tree was inferred by IQ-Tree (VT+F+I+G4 mixed model) as described in Text S1. Kva, Kvaramitovirus.

Fig. S5 mbio.00240-23-s0006.pdf Phylogenetic positions of glomeromycotinian large duamitoviruses (red letters) and 14 large duamitovirus-like viruses (ND series) with reference to the 105+94 species using the conserved RdRp domain sequences. The amino acid sequences were trimmed according to pfam05919, aligned with MAFFT (FFT-NS-i strategy), and the maximum-likelihood tree was inferred by IQ-Tree (LG+F+I+G4 mixed model) as described in Text S1. Kva, Kvaramitovirus.

Dataset S1 mbio.00240-23-s0007.xlsx Metadata of 105 and 94 additional reference species.

Dataset S2 mbio.00240-23-s0008.xlsx Metadata of large duamitovirus-like viruses in GenBank and RVMT.

Dataset S3 mbio.00240-23-s0009.xlsx Metadata of viruses similar to the 105 species in RVMT.

Dataset S4 mbio.00240-23-s0010.xlsx Putative virus genera and host taxa of the mitovirus-like viruses in the 184 libraries.

ASM does not own the copyrights to Supplemental Material that may be linked to, or accessed through, an article. The authors have granted ASM a non-exclusive, world-wide license to publish the Supplemental Material files. Please contact the corresponding author directly for reuse.
