# Supplementary material for: Structurally distinct mitoviruses: are they an ancestral lineage of the Mitoviridae exclusive to arbuscular mycorrhizal fungi (Glomeromycotina)?
Source: mBio. 2023 May 10;14(4):e00240-23. doi: 10.1128/mbio.00240-23 (PMC10470734; doi:10.1128/mbio.00240-23)
Supplement: Text S1 — Detailed materials and methods for the identification of new mitoviruses, database surveys, and phylogenetic/ecological analyses with references. [file mbio.00240-23-s0001.pdf]

Supplementary material

Structurally distinct mitoviruses: Are they an ancestral lineage of the *Mitoviridae* exclusive to arbuscular mycorrhizal fungi (Glomeromycotina)?

Tatsuhiro Ezawa, Alessandro Silvestri, Hayato Maruyama, Keitaro Tawaraya, Mei Suzuki, Yu Duan, Massimo Turina, and Luisa Lanfranco

Address correspondence to
Tatsuhiro Ezawa, tatsu@res.agr.hokudai.ac.jp
Luisa Lanfranco, luisa.lanfranco@unito.it

TEXT S1

MATERIALS AND METHODS

Identification and sequence analysis

New mitoviruses in the three glomeromycotinian fungi *Gigaspora rosea* DAOM194757, *Rhizophagus clarus* CK001, and *R. clarus* OL1 were identified by *de novo* assembling of the RNA-Seq reads from different sources as follows.

For *G. rosea* mitoviruses, the sequence reads obtained by the three RNA-Seq libraries of extraradical mycelium (SRR1979264, SRR1979265, and SRR1979266) (1) were downloaded from the Sequence Read Archive (SRA) of GenBank.

R. clarus CK001 (2) has been maintained by the open pot culture in a greenhouse and the *in vitro* monoxenic culture with the Ri T-DNA transformed roots of flax (*Linum usitatissimum* L.) purchased from the Glomeromycota In vitro Collection (http://www.mycorrhiza.be/ginco-bel/), following the method described by Becard and Fortin (3). Large subunit ribosomal RNA sequences of this strain have been deposited in GenBank under the accession nos. AB968640 – AB968645. Mycelial samples for double-stranded RNA (dsRNA) extraction were obtained as described in Ikeda et al (4). Briefly, *Lotus japonicus* MG-20 was inoculated with 1,000 spores of *R. clarus* CK001 obtained by the open culture and grown in the mesh bag-separated compartment culture system in a growth chamber for 8 weeks. Extraradical mycelia in the hyphal compartment were harvested by the wet sieving, blotted on a filter paper, weighed, and stored at -80°C until dsRNA extraction. The frozen mycelia collected from a total of 36 pots (approx. 0.2 – 0.6 g FW) were ground in the presence of liquid nitrogen in a mortar, and dsRNA was extracted, purified, and visualized by gel electrophoresis, and 100-bp-paired end sequence was performed on the Illumina HiSeq platform (Bioengineering Lab Co., Ltd., Sagamihara, Japan).

The initial culture of *R. clarus* OL1 (MAFF520078) was obtained from the NARO Genebank (https://www.gene.affrc.go.jp/index_en.php). *Brachypodium distachyon* Bd21 was inoculated with 200 spores of *R. clarus* OL1 and grown in sterilized Ando soil in a growth chamber for 26 and 40 days, and total RNA was extracted from the roots and subjected to 100-bp-paired end sequencing on the Illumina HiSeq plat form. The sequence reads were mapped to the *B. distachyon* genome and also to the *R. clarus* HR1 genome (5), and then unmapped reads were collected for searching viral sequences.

Each of the RNA-Seq reads of the three fungi was *de novo* assembled with Trinity 2.11.0., and the contigs were subjected to blastx searches against the GenBank protein database of viruses. The output was subjected to a further blastx search against the nr database (without any taxonomy limitation) and manually checked to discriminate true viral sequences from contigs of other origins. ORFs were predicted with the getorf function in EMBOSS explorer at https://www.bioinformatics.nl/cgi-bin/emboss/getorf. By these processes, five, two, and three contigs were identified as mitoviruses from *G. rosea* DAOM194757, *R. clarus* CK001, and *R. clarus* OL1, respectively (Dataset S1).

To verify that the two mitoviruses were from *R. clarus* CK001, but not from other organisms that might coexist in the open pot culture, dsRNA was extracted from about 100 spores produced in the *in vitro* monoxenic culture, purified, reverse transcribed, and amplified according to Ikeda et al. (4) using the following primer pairs: RclMV3_F/R, TGACGCGAAAAGAGTCACCA/ TCTACCCACGAGATCCGAGA; RclMV4_F/R, TCTTGCCCGTCCTGGAGATA/ TTCCAACAGGAACGGGTCTG. The PCR products were electrophoresed, cloned, and sequenced as described previously (4). About 500 bp DNA fragments were successfully amplified from the dsRNA fraction from the *in vitro* spores for each of RclMV3 and RclMV4 by RT-PCR, and their sequences were identical to those determined by the RNA-Seq (data not shown).

To verify that the three mitoviruses were from *R. clarus* OL1, but not from the host plant and other organisms, *R. clarus* OL1 was also grown in association with the different plant *Miscanthus sinensis* in a different soil (acid sulfate soil) in the mesh bag-separated compartment culture (4), and RNA was extracted from extraradical hyphae in the hyphal compartment for 100-bp-paired end RNA-Seq (10 M reads per sample). The sequence reads were mapped to the viral genome, in which more than one thousand reads were mapped to each genome of the three mitoviruses, confirmed that they are harbored by *R. clarus* OL1 (data not shown).

The conserved RdRp motif and N-terminal amino acid sequences were searched by aligning with the ClustalW algorithm implemented in MEGA X (10.1.8) (6), and conserved residues were highlighted with colors in Jalview 2.11.1.7 (7). Codon frequencies were inferred with the Codon Usage option in the Sequence Manipulation Suite (8) at the web server (http://www.bioinformatics.org/sms2/).

Database survey for large duamitovirus-like viruses

As a first step for exploring large duamitovirus-like sequences, blastp and tblastn searches were conducted against the GenBank protein and nucleotide databases of viruses using the N-terminal motif protein sequences of all the eight large duamitoviruses as queries at an E-value cutoff of $1e^{-5}$; the latter searches enabled us to identify a potential coding region, even in a different reading frame of RdRp, in viral genomes. We also conducted tblastn searches against the Mitoviridae/Unclassified datasets in the RNA Viruses in Metatranscriptomes (RVMT) database (https://riboviria.org/) for identifying those encoding the N-terminal motif. In these searches 72 and 868 sequences were identified from the GenBank and RVMT databases, respectively. From these sequences, those that encode complete RdRp of 900 aa (2,700 nt) or larger were retrieved via predicting ORF with the getorf function of EMBOSS explorer (https://www.bioinformatics.nl/cgi-bin/emboss/) and clustered with CD-HIT (9) at a criterion of 1.0 (100% aa identity), resulted in the identification of 37 and 415 sequences from the GenBank and RVMT databases (452 sequences in total) (Dataset S2). Metadata of the sequences retrieved from the RVMT database were obtained from the Metatranscriptome database in the Integrated Microbial Genomes and Microbiomes (IMG/M) at JGI (https://img.jgi.doe.gov/cgi-bin/m/main.cgi), referring to the IMG Genome (source) IDs, for mapping the locations from which the soil and root samples for metatranscriptome studies were collected. Principal component analysis (PCA) was performed with the ade4 package ver. 1.7-18 (10) in R (11), in which the 452 large duamitovirus-like sequences were further clustered at 0.95 (214 sequences in total) prior to the analysis to reduce complexity of the plot. PCA biplots were drawn with the factoextra package ver. 1.07 (https://rpkgs.datanovia.com/factoextra/index.html) in R.

Phylogenetic analysis

For phylogenetic analysis, we created three different datasets: i) all 23 (13 previously described and 10 newly identified) glomeromycotinian mitoviruses and the 105+94 reference species collections with 14 large duamitovirus-like sequences, ii) all 23 glomeromycotinian mitoviruses and the 105+94 reference species collections with 40 large duamitovirus-like sequences, and iii) all 23 glomeromycotinian mitoviruses and the 105+94 reference species with 14 large duamitovirus-like sequences in which only the conserved RdRp motif region was trimmed in reference to the standard sequences listed in pfam05919 (https://www.ncbi.nlm.nih.gov/Structure/cdd/cddsrv.cgi?uid=253459). All the 14 large duamitovirus-like sequences were selected from the those identified in the RVMT database (Dataset S2); a half of them use UGA codons for Trp more than 10%, and the other half was randomly selected from those that use no UGA codon. The 40 large duamitovirus-like sequences consisted of 17 and 9 sequences randomly selected from those identified in the GenBank database and the RVMT database, respectively, in addition to the 14 sequences (Dataset S2).

Multiple sequence alignment was performed with MAFFT ver. 7 (12) either with the L-INS-i strategy for full-length sequences or with the automatic selection mode for the trimmed sequences at the web server (https://mafft.cbrc.jp/alignment/server/) and Clustal Omega (13) at the EMBL-EBI server (https://www.ebi.ac.uk/Tools/msa/). The maximum likelihood trees were inferred with W-IQ-TREE 1.6.11 (14) at the web server (http://iqtree.cibiv.univie.ac.at/) with the "Find best and apply" mode of ModelFinder (15), in which reliability for internal branch was assessed using the UltraFast Bootstrapping (1000 replicates) (16). Two narnaviruses and two leviviruses were employed as outgroup (Dataset S1). Only the trees in which branch-support values at the major nodes that separate the genera are higher than 60% are presented.

Niche/habitat analysis of large duamitovirus-like viruses

Prior to searching sequences similar to the 105 reference species, those that have information about geographic location, ecosystem, habitat, and sample type in the "*Mitoviridae*" and "Unclassified" datasets in the RVMT database were extracted (23,410 and 37,980 sequences from the *Mitoviridae* and Unclassified data, respectively) and combined (61,390 sequences in total). Against these sequences, tblastn searches were performed using the 105 reference species (excluding glomeromycotinian large duamitoviruses) as queries, and top 20 sequences showing similarity to each of the 105 species were retrieved and clustered at 100% nt identity. From them, those that encode complete protein (between start and stop codons) of 500 aa or longer with the translation table 4 (yeast mitochondria) were extracted and clustered at 100% identity, by which 1,052 sequences in total were obtained (Dataset S3). These 1,052 viral sequences were grouped by putative virus genera and host phyla/domains defined in reference to the most similar reference species and also by sampling location, ecosystem, and sample type, and a mitovirus-community dataset was constructed, together with the 451 large duamitovirus-like sequences. Non-metric multidimensional scaling (NMDS) and correlation analysis of the NMDS1 and 2 scores of species (genus-phylum scores in this case) with the frequencies of the ecosystem and sample-type data in the individual studies were performed with the vegan ver. 2.6-4 (17) in R using Bray-Curtis dissimilarity index as a distance metric.

To infer putative hosts of mitovirus-like viruses co-existed with the large duamitovirus-like viruses in the sequencing libraries, the sequences that encode complete protein of 500 aa or longer were retrieved from the 61,390 sequences in the "*Mitoviridae*" and "Unclassified" datasets in the RVMT database and clustered the protein sequences with CD-HIT at 100% identity, yielded 26,003 non-redundant mitovirus-like sequences from 2,888 libraries, including the 184 libraries that contain 8,361 non-

redundant sequences from which the 415 large duamitoviruses were identified (samples/sequencing libraries are identified by IMG Genome ID in Dataset S4). Then the 8,361 sequences were subjected to blastp searches against the 105 reference species and all (non-large) glomeromycotinian mitoviruses at an E-value cutoff of $1e^{-30}$, which yielded 4,928 sequences, and their host taxa (phyla/kingdom) and virus genera were assigned in reference to the top hit reference species. Significance of correlation between the numbers of large duamitovirus-like sequences and those of the other mitovirus-like sequences was assessed by Student's *t*-test.

REFERENCES

1. Tang N, San Clemente H, Roy S, Bécard G, Zhao B, Roux C. 2016. A survey of the gene repertoire of *Gigaspora rosea* unravels conserved features among Glomeromycota for obligate biotrophy. *Front Microbiol* 7:233.
2. Sato T, Hachiya S, Inamura N, Ezawa T, Cheng W, Tawaraya K. 2019. Secretion of acid phosphatase from extraradical hyphae of the arbuscular mycorrhizal fungus *Rhizophagus clarus* is regulated in response to phosphate availability. *Mycorrhiza* 29:599-605.
3. Bécard G, Fortin JA. 1988. Early events of vesicular-arbuscular mycorrhiza formation on Ri T-DNA transformed roots. *New Phytol* 108:211-218.
4. Ikeda Y, Shimura H, Kitahara R, Masuta C, Ezawa T. 2012. A novel virus-like double-stranded RNA in an obligate biotroph arbuscular mycorrhizal fungus: a hidden player in mycorrhizal symbiosis. *Mol Plant-Microbe Interact* 25:1005-1012.
5. Kobayashi Y, Maeda T, Yamaguchi K, Kameoka H, Tanaka S, Ezawa T, Shigenobu S, Kawaguchi M. 2018. The genome of *Rhizophagus clarus* HR1 reveals a common genetic basis for auxotrophy among arbuscular mycorrhizal fungi. *BMC Genomics* 19:465.
6. Kumar S, Stecher G, Li M, Knyaz C, Tamura K. 2018. MEGA X: Molecular evolutionary genetics analysis across computing platforms. *Mol Biol Evol* 35:1547-1549.
7. Waterhouse AM, Procter JB, Martin DMA, Clamp M, Barton GJ. 2009. Jalview Version 2—a multiple sequence alignment editor and analysis workbench. *Bioinformatics* 25:1189-1191.
8. Stothard P. 2000. The Sequence Manipulation Suite: JavaScript programs for analyzing and formatting protein and DNA sequences. *BioTechniques* 28:1102-1104.
9. Fu L, Niu B, Zhu Z, Wu S, Li W. 2012. CD-HIT: accelerated for clustering the next-generation sequencing data. *Bioinformatics* 28:3150-3152.
10. Dray S, Dufour A-B. 2007. The ade4 Package: Implementing the duality diagram for ecologists. *J Statistic Software* 22:1-20.

11. R_Core_Team. 2021. R: A language and environment for statistical computing, R Foundation for Statistical Computing, Vienna, Austria. http://www.R-project.org/.
12. Katoh K, Rozewicki J, Yamada KD. 2017. MAFFT online service: multiple sequence alignment, interactive sequence choice and visualization. *Briefing Bioinformatics* 20:1160-1166.
13. Sievers F, Higgins DG. 2018. Clustal Omega for making accurate alignments of many protein sequences. *Protein Sci* 27:135-145.
14. Trifinopoulos J, Nguyen L-T, von Haeseler A, Minh BQ. 2016. W-IQ-TREE: a fast online phylogenetic tool for maximum likelihood analysis. *Nucl Acid Res* 44:W232-W235.
15. Kalyaanamoorthy S, Minh BQ, Wong TKF, von Haeseler A, Jermiin LS. 2017. ModelFinder: fast model selection for accurate phylogenetic estimates. *Nat Meth* 14:587-589.
16. Hoang DT, Chernomor O, von Haeseler A, Minh BQ, Vinh LS. 2017. UFBoot2: Improving the ultrafast bootstrap approximation. *Mol Biol Evol* 35:518-522.
17. Oksanen J, Simpson G, Blanchet F, Kindt R, Legendre P, Minchin P, O'Hara R, Solymos P, Stevens M, Szoecs E, Wagner H, Barbour M, Bedward M, Bolker B, Borcard D, Carvalho G, Chirico M, De Caceres M, Durand S, Evangelista H, FitzJohn R, Friendly M, Furneaux B, Hannigan G, Hill M, Lahti L, McGlinn D, Ouellette M, Ribeiro Cunha E, Smith T, Stier A, Ter Braak C, Weedon J. 2022. vegan: Community Ecology Package. R package version 2.6-4., http://CRAN.R-project.org/package=vegan.
