# Supplementary material for: Structurally distinct mitoviruses: are they an ancestral lineage of the Mitoviridae exclusive to arbuscular mycorrhizal fungi (Glomeromycotina)?
Source: mBio. 2023 May 10;14(4):e00240-23. doi: 10.1128/mbio.00240-23 (PMC10470734; doi:10.1128/mbio.00240-23)
Supplement: Fig. S1 — Multiple alignment of the conserved amino acid motifs in mitovirus RNA-dependent RNA polymerase in the newly identified glomeromycotinian mitoviruses. The RdRp sequences from the 10 newly identified viruses (indicated with asterisks and bold letters), previously described 5 glomeromycotinian viruses (bold letters), Duamitovirus crpa1, and Duamitovirus soch1 (Dataset S1) were aligned by Clustal W implemented in MEGA X. Aligned amino acid residues are highlighted with colors in Jalview, and the positions of motifs are indicated by black lines. [file mbio.00240-23-s0002.pdf]

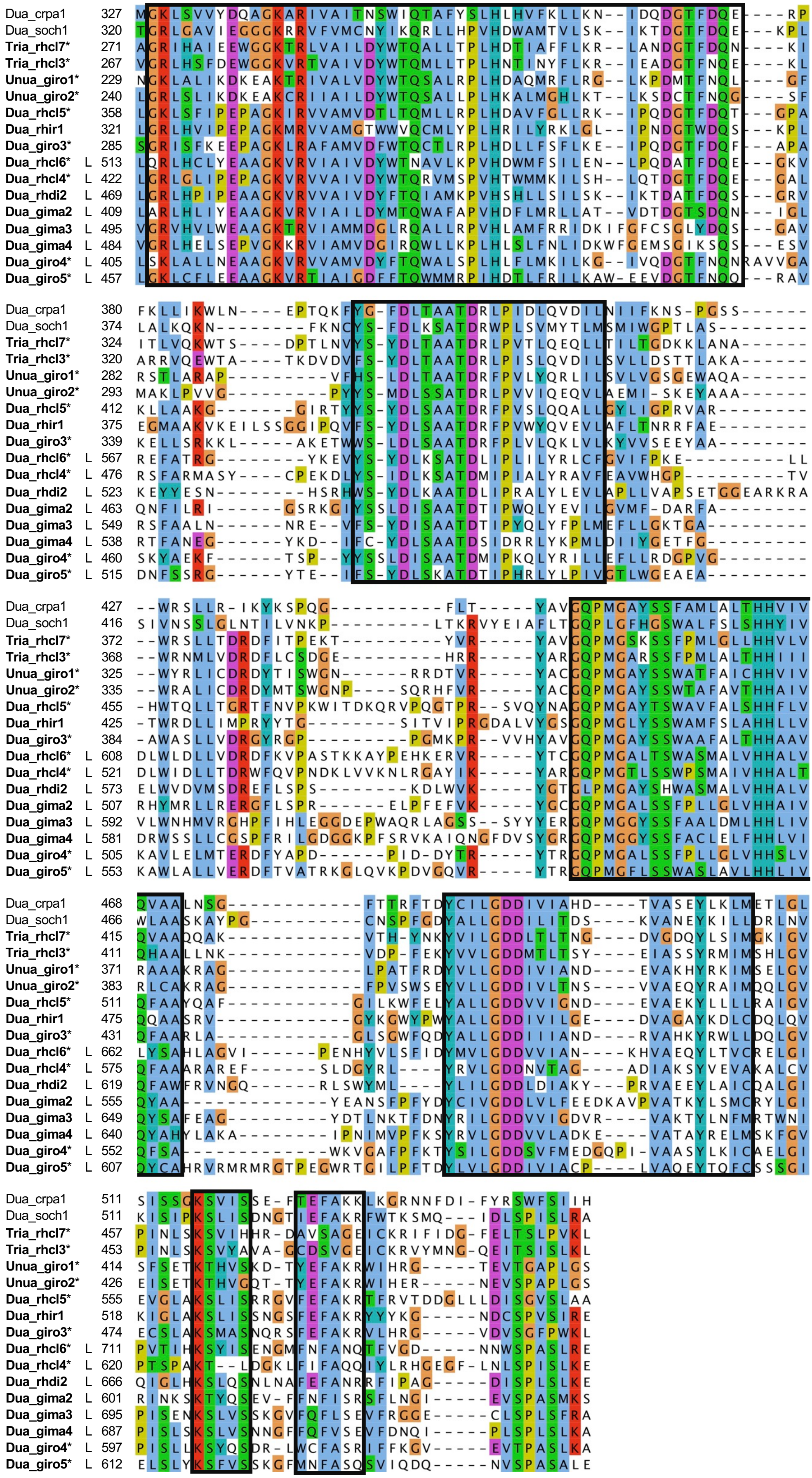

Dua_crpa1 327
Dua_soch1 320
Tria_rhcl7* 271
Tria_rhcl3* 267
Unua_giro1* 229
Unua_giro2* 240
Dua_rhcl5* 358
Dua_rhir1 321
Dua_giro3* 285
Dua_rhcl6* L 513
Dua_rhcl4* L 422
Dua_rhdi2 L 469
Dua_gima2 L 409
Dua_gima3 L 495
Dua_gima4 L 484
Dua_giro4* L 405
Dua_giro5* L 457
Dua_crpa1 380
Dua_soch1 374
Tria_rhcl7* 324
Tria_rhcl3* 320
Unua_giro1* 282
Unua_giro2* 293
Dua_rhcl5* 412
Dua_rhir1 375
Dua_giro3* 339
Dua_rhcl6* L 567
Dua_rhcl4* L 476
Dua_rhdi2 L 523
Dua_gima2 L 463
Dua_gima3 L 549
Dua_gima4 L 538
Dua_giro4* L 460
Dua_giro5* L 515
Dua_crpa1 427
Dua_soch1 416
Tria_rhcl7* 372
Tria_rhcl3* 368
Unua_giro1* 325
Unua_giro2* 335
Dua_rhcl5* 455
Dua_rhir1 425
Dua_giro3* 384
Dua_rhcl6* L 608
Dua_rhcl4* L 521
Dua_rhdi2 L 573
Dua_gima2 L 507
Dua_gima3 L 592
Dua_gima4 L 581
Dua_giro4* L 505
Dua_giro5* L 553
Dua_crpa1 468
Dua_soch1 466
Tria_rhcl7* 415
Tria_rhcl3* 411
Unua_giro1* 371
Unua_giro2* 383
Dua_rhcl5* 511
Dua_rhir1 475
Dua_giro3* 431
Dua_rhcl6* L 662
Dua_rhcl4* L 575
Dua_rhdi2 L 619
Dua_gima2 L 555
Dua_gima3 L 649
Dua_gima4 L 640
Dua_giro4* L 552
Dua_giro5* L 607
Dua_crpa1 511
Dua_soch1 511
Tria_rhcl7* 457
Tria_rhcl3* 453
Unua_giro1* 414
Unua_giro2* 426
Dua_rhcl5* 555
Dua_rhir1 518
Dua_giro3* 474
Dua_rhcl6* L 711
Dua_rhcl4* L 620
Dua_rhdi2 L 666
Dua_gima2 L 601
Dua_gima3 L 695
Dua_gima4 L 687
Dua_giro4* L 597
Dua_giro5* L 612
