# Supplementary figures and images for: Structurally distinct mitoviruses: are they an ancestral lineage of the Mitoviridae exclusive to arbuscular mycorrhizal fungi (Glomeromycotina)?
Source: mBio. 2023 May 10;14(4):e00240-23. doi: 10.1128/mbio.00240-23 (PMC10470734; doi:10.1128/mbio.00240-23)

**N-terminal  
motif**

**GDD**

*Gi. rosea* MV5

ND\_033221

ND\_045848

ND\_049966

ND\_052336

ND\_133833

ND\_172758

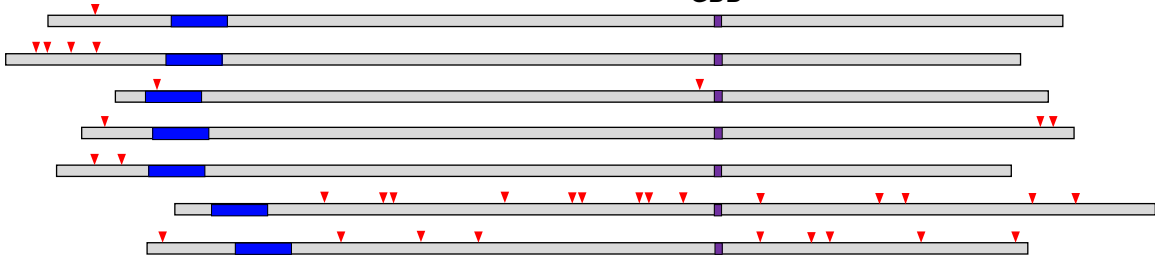

Supplement: Fig. S2 — Distribution of UGA codon for Trp (red arrow heads) in the RdRps of glomeromycotinian large duamitovirus (G. rosea MV5) and six large duamitovirus-like viruses employed for the phylogenetic analyses (Fig. S3 to S7). The RdRps are aligned at the GDD motif (purple boxes), and the N-terminal motif is indicated by blue boxes. [file mbio.00240-23-s0003.pdf]

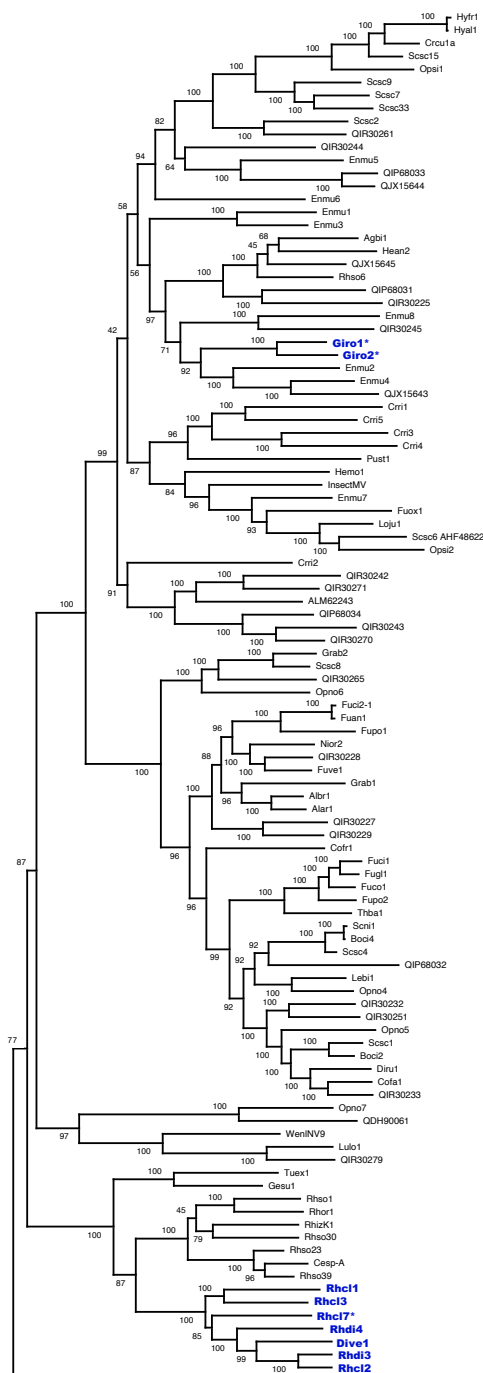

Unamitovirus

Kva

Triamitovirus

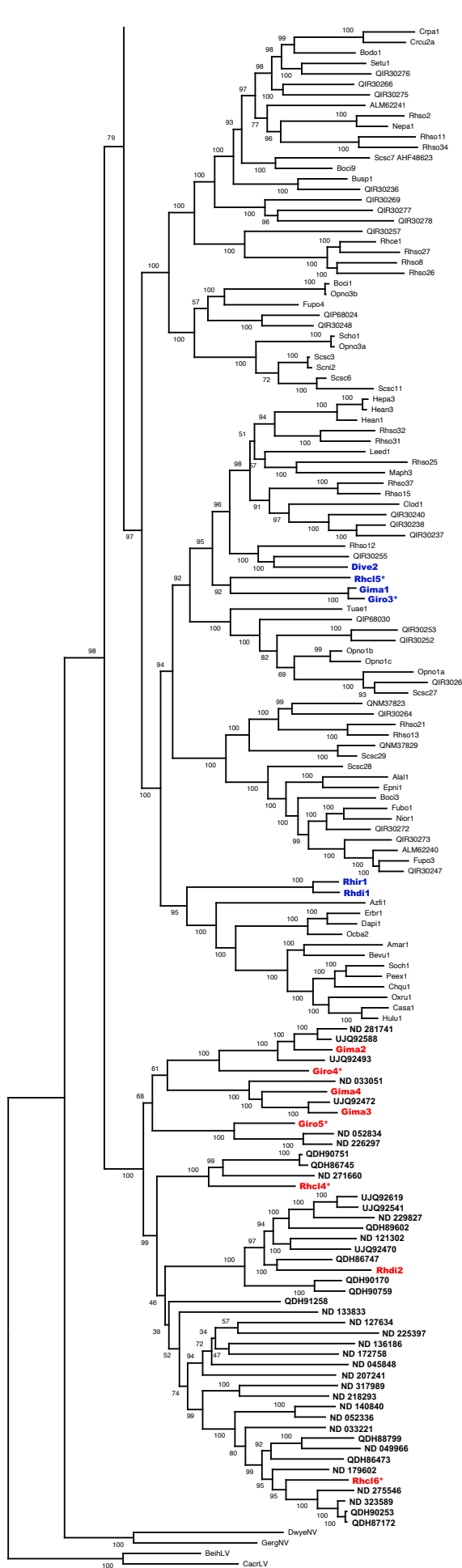

Duamitovirus

Large Duamitovirus

Supplement: Fig. S3 — Phylogenetic positions of glomeromycotinian large duamitoviruses (red letters) and 40 large duamitovirus-like viruses (ND series) with reference to the 105+94 species (B). The full-length amino acid sequences of RdRp were aligned with MAFFT (L-INS-i strategy), and the maximum-likelihood tree was inferred by IQ-Tree (VT+F+I+G4 mixed model) as described in Text S1. Kva, Kvaramitovirus. [file mbio.00240-23-s0004.pdf]

Triamivovirus

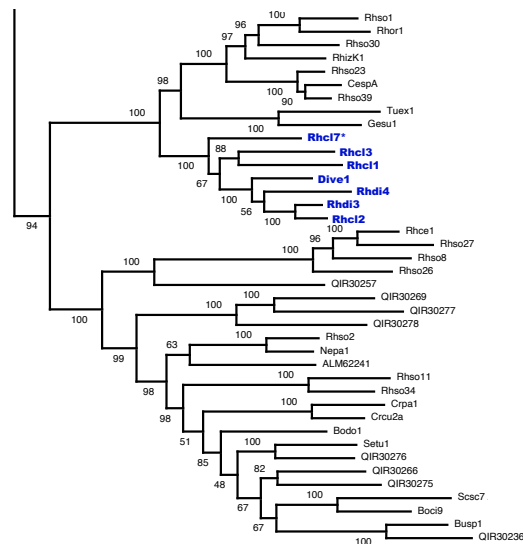

Duamivovirus

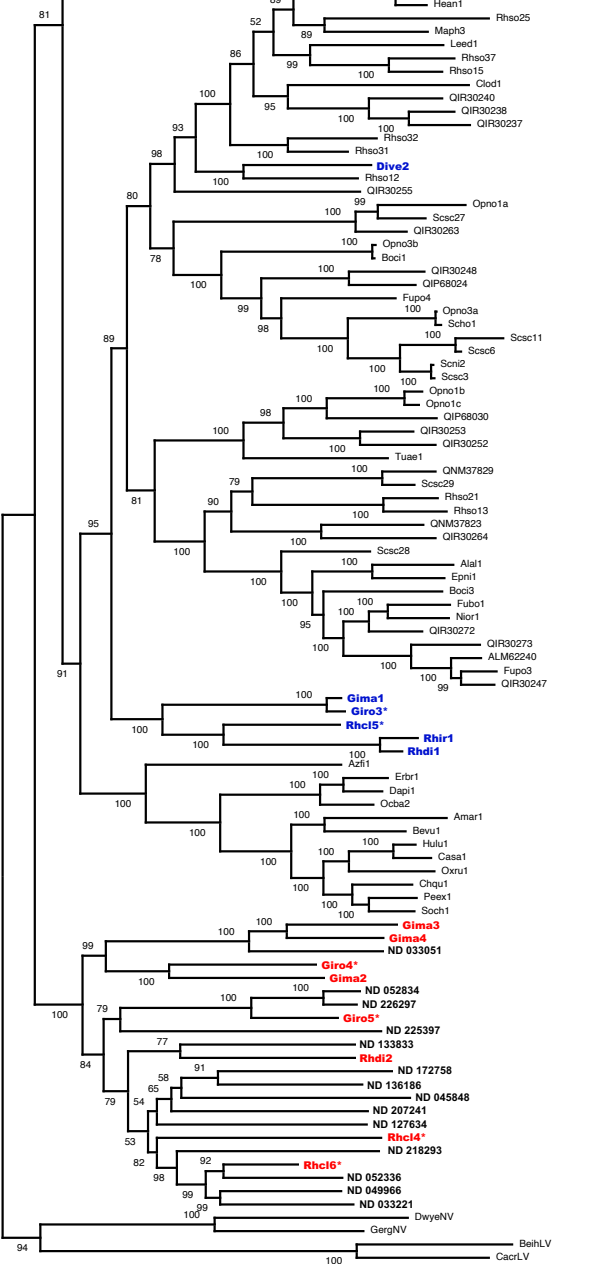

Large Duamivovirus

Unuamivovirus

Kva

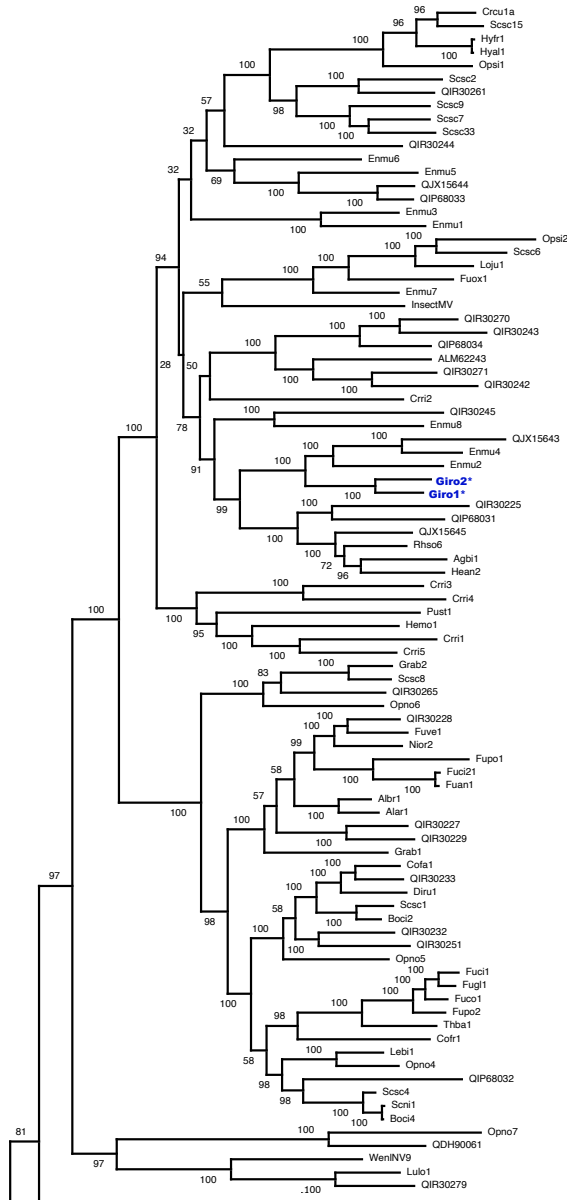

Supplement: Fig. S5 — Phylogenetic positions of glomeromycotinian large duamitoviruses (red letters) and 14 large duamitovirus-like viruses (ND series) with reference to the 105+94 species using the conserved RdRp domain sequences. The amino acid sequences were trimmed according to pfam05919, aligned with MAFFT (FFT-NS-i strategy), and the maximum-likelihood tree was inferred by IQ-Tree (LG+F+I+G4 mixed model) as described in Text S1. Kva, Kvaramitovirus. [file mbio.00240-23-s0006.pdf]
