# Supplementary material for: Structurally distinct mitoviruses: are they an ancestral lineage of the Mitoviridae exclusive to arbuscular mycorrhizal fungi (Glomeromycotina)?
Source: mBio. 2023 May 10;14(4):e00240-23. doi: 10.1128/mbio.00240-23 (PMC10470734; doi:10.1128/mbio.00240-23)
Supplement: Fig. S4 — Phylogenetic positions of glomeromycotinian large duamitoviruses (red letters) and 14 large duamitovirus-like viruses (ND series) with reference to the 105+94 species. The full-length amino acid sequences of RdRp were aligned with Clustal Omega, and the maximum-likelihood tree was inferred by IQ-Tree (VT+F+I+G4 mixed model) as described in Text S1. Kva, Kvaramitovirus. [file mbio.00240-23-s0005.pdf]

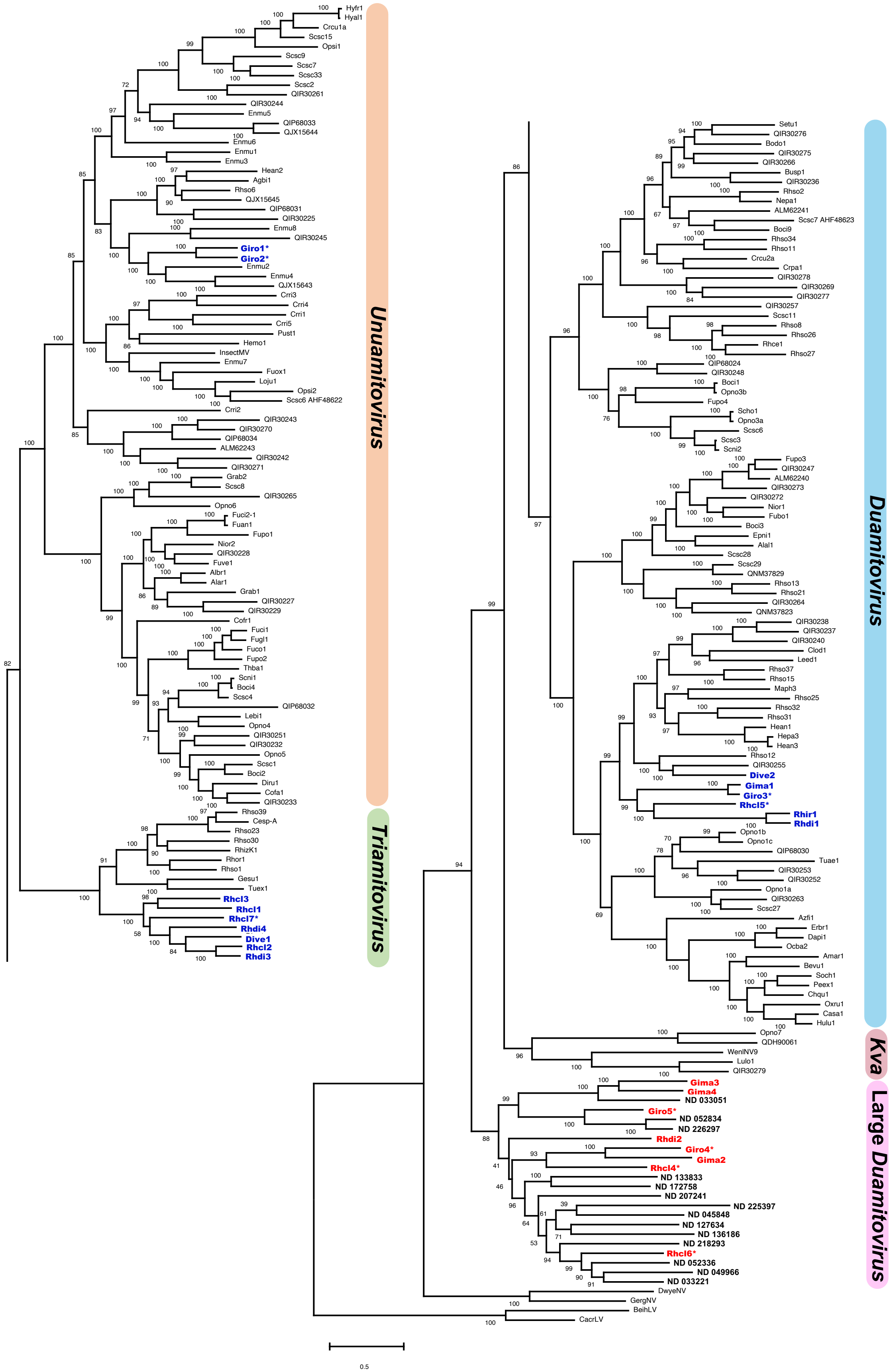
Unuamitovirus
Triamitovirus
Duamitovirus
Kva
Large Duamitovirus
Hyfr1
Hyal1
Crcu1a
Scsc15
Opsi1
Scsc9
Scsc7
Scsc33
Scsc2
QIR30261
QIR30244
Enmu5
QIP68033
QJX15644
Enmu6
Enmu1
Enmu3
Hean2
Agbi1
Rhso6
QJX15645
QIP68031
QIR30225
Enmu8
QIR30245
Giro1*
Giro2*
Enmu2
Enmu4
QJX15643
Crri3
Crri4
Crri1
Crri5
Pust1
Hemo1
InsectMV
Enmu7
Fuox1
Loju1
Opsi2
Scsc6 AHF48622
Crri2
QIR30243
QIR30270
QIP68034
ALM62243
QIR30242
QIR30271
Grab2
Scsc8
QIR30265
Opno6
Fuci2-1
Fuan1
Fupo1
Nior2
QIR30228
Fuve1
Albr1
Alar1
Grab1
QIR30227
QIR30229
Cofr1
Fuci1
Fugl1
Fuco1
Fupo2
Thba1
Scni1
Boci4
Scsc4
QIP68032
Lebi1
Opno4
QIR30251
QIR30232
Opno5
Scsc1
Boci2
Diru1
Cofa1
QIR30233
Rhso39
Cesp-A
Rhso23
Rhso30
RhizK1
Rhor1
Rhso1
Gesu1
Tuex1
Rhcl3
Rhcl1
Rhcl7*
Rhdi4
Dive1
Rhcl2
Rhdi3
Setu1
QIR30276
Bodo1
QIR30275
QIR30266
Busp1
QIR30236
Rhso2
Nepa1
ALM62241
Scsc7 AHF48623
Boci9
Rhso34
Rhso11
Crcu2a
Crpa1
QIR30278
QIR30269
QIR30277
QIR30257
Scsc11
Rhso8
Rhso26
Rhce1
Rhso27
QIP68024
QIR30248
Boci1
Opno3b
Fupo4
Scho1
Opno3a
Scsc6
Scsc3
Scni2
Fupo3
QIR30247
ALM62240
QIR30273
QIR30272
Nior1
Fubo1
Boci3
Epni1
Alal1
Scsc28
Scsc29
QNM37829
Rhso13
Rhso21
QIR30264
QNM37823
QIR30238
QIR30237
QIR30240
Clod1
Leed1
Rhso37
Rhso15
Maph3
Rhso25
Rhso32
Rhso31
Hean1
Hepa3
Hean3
Rhso12
QIR30255
Dive2
Gima1
Giro3*
Rhcl5*
Rhir1
Rhdi1
Opno1b
Opno1c
QIP68030
Tuae1
QIR30253
QIR30252
Opno1a
QIR30263
Scsc27
Azfi1
Erbr1
Dapi1
Ocba2
Amar1
Bevu1
Soch1
Peex1
Chqu1
Oxru1
Casa1
Hulu1
Opno7
QDH90061
WenlNV9
Lulo1
QIR30279
Gima3
Gima4
ND 033051
Giro5*
ND 052834
ND 226297
Rhdi2
Giro4*
Gima2
Rhcl4*
ND 133833
ND 172758
ND 207241
ND 225397
ND 045848
ND 127634
ND 136186
ND 218293
Rhcl6*
ND 052336
ND 049966
ND 033221
DwyeNV
GergNV
BeihLV
CacrLV
0.5
